# Can Acupuncture Treatment Be Double-Blinded? An Evaluation of Double-Blind Acupuncture Treatment of Postoperative Pain

**DOI:** 10.1371/journal.pone.0119612

**Published:** 2015-03-06

**Authors:** Lene Vase, Sara Baram, Nobuari Takakura, Miho Takayama, Hiroyoshi Yajima, Akiko Kawase, Lars Schuster, Ted J. Kaptchuk, Søren Schou, Troels Staehelin Jensen, Robert Zachariae, Peter Svensson

**Affiliations:** 1 Department of Psychology and Behavioural Sciences, Aarhus University, Aarhus, Denmark; 2 Danish Pain Research Center, Aarhus University Hospital, Aarhus, Denmark; 3 Section of Clinical Oral Physiology, Department of Dentistry, Aarhus University, Aarhus, Denmark; 4 Section of Oral and Maxillofacial Surgery and Oral Pathology, Department of Dentistry, Aarhus University, Aarhus, Denmark; 5 Department of Acupuncture and Moxibustion, Tokyo Ariake University of Medical and Health Sciences, Tokyo, Japan; 6 The Acupuncture Academy, Copenhagen and Aarhus, Denmark; 7 Beth Israel Deaconess Medical Center, Harvard Medical School, Boston, Massachusetts, United States of America; 8 Department of Oncology, Aarhus University Hospital, Aarhus, Denmark; University of St Andrews, UNITED KINGDOM

## Abstract

Blinding protects against bias but the success of blinding is seldom assessed and reported in clinical trials including studies of acupuncture where blinding represents a major challenge. Recently, needles with the potential for double-blinding were developed, so we tested if acupuncture can be double-blinded in a randomized study of sixty-seven patients with acute pain ≥ 3 (0-10 scale following third molar removal) who received active acupuncture with a penetrating needle or placebo acupuncture with a non-penetrating needle. To test if acupuncture was administered double-blind, patients and acupuncturists were asked about perceived treatment allocation at the end of the study. To test if there were clues which led to identification of the treatment, deep dull pain associated with needle application and rotation (termed “*de qi*” in East Asian medicine), and patients’ pain levels were assessed. Perceived treatment allocation depended on actual group allocation (p < 0.015) for both patients and acupuncturists, indicating that the needles were not successful in double-blinding. Up to 68% of patients and 83% of acupuncturists correctly identified the treatment, but for patients the distribution was not far from 50/50. Also, there was a significant interaction between actual or perceived treatment and the experience of *de qi* (p = 0.027), suggesting that the experience of *de qi* and possible non-verbal clues contributed to correct identification of the treatment. Yet, of the patients who perceived the treatment as active or placebo, 50% and 23%, respectively, reported *de qi*. Patients’ acute pain levels did not influence the perceived treatment. In conclusion, acupuncture treatment was not fully double-blinded which is similar to observations in pharmacological studies. Still, the non-penetrating needle is the only needle that allows some degree of practitioner blinding. The study raises questions about alternatives to double-blind randomized clinical trials in the assessment of acupuncture treatment.

## Introduction

The success of blinding ought to be reported in clinical trials [1]. However, most studies do not report the success of blinding, and even fewer studies apply adequate tests for double-blinding [2–4], thereby hampering the validity of the trial and questioning the inferred conclusions. Furthermore, a systematic review has indicated that blinding is reported less often in non-pharmacological than pharmacological pain trials, even when blinding was judged feasible, which non-pharmacological trials were less often [3].

Several attempts have been made to conduct blinded, randomized clinical trials of the often used non-pharmacological treatment acupuncture [5–8]. However, most types of placebo acupuncture cannot be blinded for the acupuncturist [9,10], so double-blinding has been difficult. Lately, active and placebo acupuncture needles that appear to have the potential to be double-blinded have been developed. In short, the penetrating needle used for active acupuncture penetrates the skin, whereas the matched non-penetrating needle used for placebo acupuncture only presses against the skin without penetration. Previous studies have shown that healthy volunteers [11,12,13], and in most instances also experienced acupuncturists [11–15], were unable to correctly identify penetrating from non-penetrating needles. Still, so far no randomized clinical trial involving patients has been conducted to systematically investigate if acupuncture treatment can be double-blinded.

To test if treatments are double-blinded, it is important to directly ask both the patient and the healthcare provider, in this case the acupuncturist, if they can identify the treatment allocation [16,2,4]. Also, it is helpful to assess if there are clues that may help the patient and the acupuncturist to identify the treatment allocation correctly [9,15,16]. *De qi* is the Chinese expression for describing the sensation of acupuncture needling and includes feelings of distention, heaviness, tingling, numbness, or deep dull pain associated with the needle application and rotation [17,18]. Importantly, both the patient and the practitioner may experience *de qi* [18]. Typically, the acupuncturist would perceive *de qi* as heaviness or tenseness about the needle he or she is stimulating, and the patient would perceive *de qi* as soreness, numbness, heaviness, and distention at the site of the needle placement in response to being punctured, although these sensations may spread to other parts of the body as well [18]. *De qi* has been related to efficacy and may therefore influence the perception of whether active or placebo acupuncture is given [18]. Also, in a clinical trial setting, the patients’ experience of pain and possibly their expression of pain could influence their perception of the treatment allocation [9]. Thus, it seems relevant to assess these two factors.

As part of a recently published randomized clinical trial [19], patients who had undergone surgical removal of mandibular third molars received active acupuncture with a penetrating needle or placebo acupuncture with a non-penetrating needle. Neither the patients nor the acupuncturist were informed who received active or placebo acupuncture. The aims of the present study were to test:

1. Can patients and acupuncturists, respectively, identify whether active or placebo acupuncture treatment is given?
2. Does the experience of *de qi* and the patients’ level of pain influence the identification of the treatment allocation?

## Participants and Methods

### Larger trial

The current study is part of a larger recently published study [19]. In the larger trial, 101 patients who developed pain of ≥ 3 on a visual analogue scale (VAS, 0–10) after third molar surgery were randomized to receive active acupuncture (penetrating needles), placebo acupuncture (non-penetrating needles), or no treatment for 30 minutes. The patients’ perception of the treatment (active or placebo) and expected pain levels (VAS) were assessed before and halfway through the treatment. Looking at the actual treatment allocation, there was no specific effect of active acupuncture (difference in pain levels between active and placebo acupuncture; p = 0.240), but there was a large and significant non-specific effect of placebo acupuncture (difference in pain levels between placebo acupuncture and no treatment; p <0.001). Interestingly, however, looking at perceived treatment allocation, there was a significant effect of acupuncture (difference in pain levels between perceived active and perceived placebo acupuncture p <0.001), indicating that patients who believed they received active acupuncture had significantly lower pain levels than those who believed they received placebo acupuncture.

### Patients and Surgical Treatment

Seventy patients who were referred for surgical removal of one mandibular third molar at the Section of Oral and Maxillofacial Surgery and Oral Pathology, Department of Dentistry, Aarhus University, Aarhus, Denmark, from September 2008 to July 2009 participated in the study. Only 67 of the 101 patients included in the larger trial were eligible for the current study, since it did not include a no-treatment condition.

In order to be included in the study, the patients had to have pain ≥ 3 on a mechanical visual analog scale (M-VAS, 0–10) 4 hours after surgery [20], be ≥ 18 years of age and in good health defined as risk groups I (normal healthy patients), II (patients with mild systemic disease), or III (patients with severe systemic disease that is not incapacitating) according to the American Society of Anesthesiologist (ASA) [21]. The indications for surgical removal of one semi-impacted mandibular third molar involving bone removal were: 1) recurring episodes of pericoronitis (≥ 2 episodes), 2) caries or resorption of the second molar (distal surface), 3) unrestorable caries of the third molar, 4) progressive periodontitis of the second molar (distal surface) or third molar, or 5) other pathologic conditions related to the third molar.

Patients were excluded if they 1) had previous experience with acupuncture, 2) experienced pain below 3 (M-VAS, 0–10) 4 hours after surgery as a VAS score of 3 is considered the demarcation between mild (< 3) and moderate pain (> 3) [20], 3) had to take rescue medication during the study period, 4) had a pain disorder that might interfere with the measurement of pain, 5) were medicated 24 hours before the experiment, 6) were pregnant or breastfeeding, or 7) suffered from medical or psychiatric disorders that prevented them from participating in the study.

Prior to surgery, the patients received local analgesia with lidocaine (20 mg/ml) with adrenaline (12.5 μg/ml) (Xyloplyin Dental Adrenalin, Dentsply, Surrey, England). The standard dose was 2 ml for the inferior alveolar and lingual nerves, 1 ml for the buccal nerve, and 1 ml for infiltration buccally. If necessary, additional local analgesia was provided during the surgical procedure and the amount (ml) of additional analgesia was registered. The third molar was removed using a standardized surgical approach. In brief, a full-thickness mucoperiosteal flap was elevated, and buccal/distal bone was removed with a burr under sterile saline irrigation. The tooth was removed in one piece or several pieces after sectioning with a burr, also under sterile saline irrigation. Inflammatory tissue and sharp bony edges were removed before meticulous irrigation of the socket and the operation field. Finally, the flap was repositioned and sutured using 2–3 resorbable sutures (Vicryl 4–0, Ethicon, Norderstedt, Germany). Most teeth (96%) were removed by dental students under the supervision of oral and maxillofacial surgeons or dentists with special training in oral surgery. Few teeth (4%) were removed by oral and maxillofacial surgeons or dentists with special training in oral surgery.

### Ethics statement

All patients received oral and written information about the study and gave signed informed consent. They were compensated with 70€ (500 DKK) for participating in the study. The study was approved by the local Ethical Committee for the Central Denmark Region [M-20070270].

### Acupuncturists

Acupuncturists trained at the Aarhus Acupuncture Academy, Denmark, licensed as Registered Alternative Practitioners and having at least 5 years of experience practicing acupuncture performed the acupuncture treatments. Four acupuncturists performed the majority of the treatment session (63 out of 67) and two acupuncturists performed the remaining four sessions. The acupuncturists performed acupuncture according to the Chinese tradition where needle rotation is considered a central part of the acupuncture, as this is the standard procedure in Denmark. Prior to the study, the acupuncturists, the research assistant (SB), and the principal investigator (LV) met to agree on and practice standardized ways of interacting with the patients [22]. Inspired by Kaptchuk and colleagues [22], each interaction was structured according to content (3 points) and style (5 points). Content included a presentation of the acupuncturist including his/her experience with acupuncture as well as questions concerning the patients’ current symptoms and previous experiences with dental pain. Style included a warm friendly manner, active listening, expression of empathy, thought, and full silence while feeling the pulse and communication of confidence and positive expectation (“acupuncture has been shown to reduce dental pain”). This approach is agreement with a prototype of an ideal healthcare interaction [23].

### Active acupuncture

**Fig 1:**
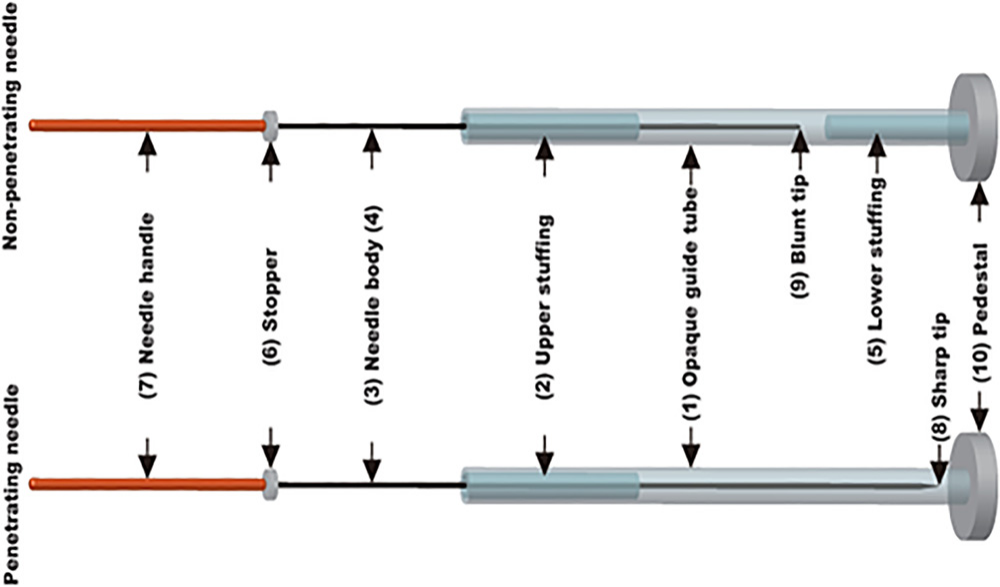
Active and placebo needles. Each needle assembly comprises an opaque guide tube (1) and upper stuffing (2) to provide resistance to the needle body during its passage through the guide tube. The body of the penetrating needle (3) is longer than the guide tube by an amount equal to the insertion depth, but the body of the non-penetrating needle (4) is only long enough to allow its blunt tip to press against the skin when the needle body is advanced to its limit. The non-penetrating needle contains stuffing at the bottom as well (5) to give a sensation similar to that of skin puncture and tissue penetration. Both needles have a stopper (6) that prevents the needle handle (7) from advancing further when the sharp tip of the penetrating needle (8) or the blunt tip of the non-penetrating needle (9) reaches the specified position. The pedestal (10) on each needle is adhesive, allowing it to adhere firmly to the skin surface. The diameter of the needles used in this study was 0.16 mm.

Active acupuncture treatment was performed with penetrating needles (Hanada College, Japan School of Acupuncture, Tokyo, Japan) [13]. The needle assembly comprised an opaque guide tube and upper stuffing to provide resistance to the needle body during its passage through the guide tube (Fig. 1). The body of the penetrating needle was longer than the guide tube by an amount equal to the insertion depth. The needle had a stop that prevented it from advancing further when the sharp tip of the penetrating needle reached the specified insertion depth of 10 mm. The pedestal on each needle was adhesive, allowing it to adhere firmly to the skin surface. The diameter of the needle was 0.16 mm.

### Placebo Acupuncture

Placebo acupuncture treatment was performed with non-penetrating needles with a similar appearance as the penetrating needles (Hanada College, Japan School of Acupuncture, Tokyo, Japan) [13]. The needle assembly comprised an opaque guide tube and upper and lower stuffing to provide resistance to the needle body during its passage through the guide tube (Fig. 1). The body of the non-penetrating needle was only long enough to allow its blunt tip to press against the skin when the needle body was advanced to its limit. The needle had a stop that prevented it from advancing further when the blunt tip of the non-penetrating needle reached the specified position. The pedestal on each needle was adhesive, allowing it to adhere firmly to the skin surface. The diameter of the needle was 0.16 mm.

### Acupuncture Points

**Fig 2:**
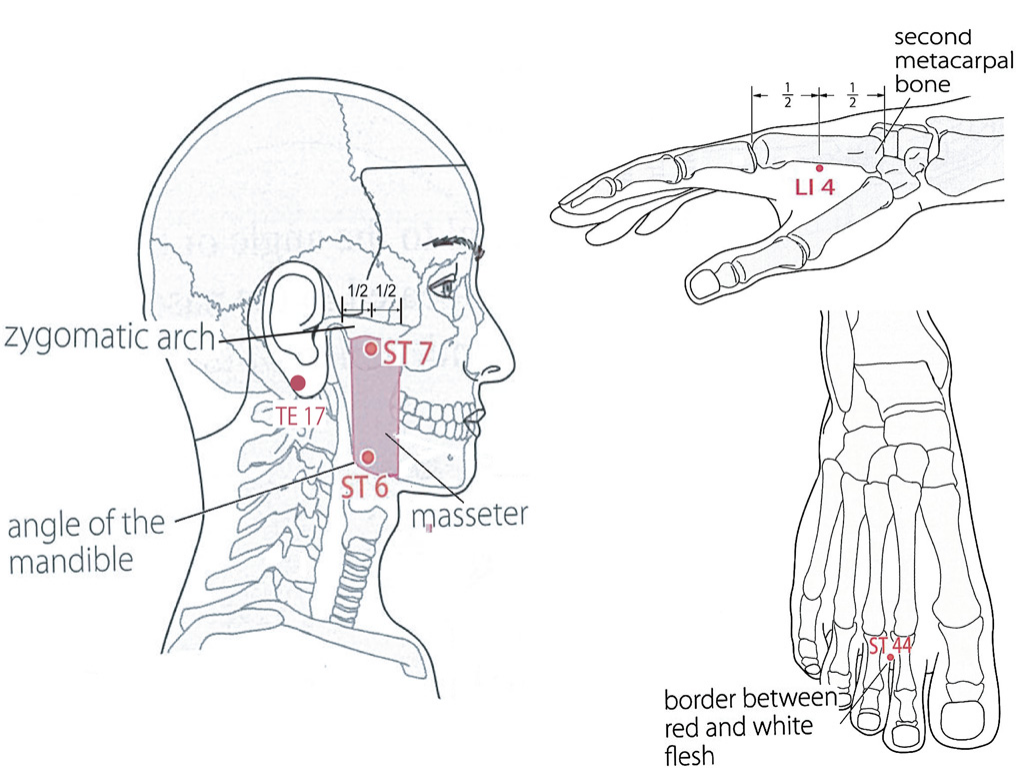
Acupuncture points. The five acupuncture points.

The needles were applied in the following positions: ST44, LI4, ST7, ST6, and TE 17 (Fig. 2). The acupuncture points LI4, ST7, ST6, and TE17 have previously been used in studies on dental pain following surgical removal of mandibular third molars [24], and ST44 has been considered for toothache and facial pain [25–27]. All acupuncturists agreed with the selection of acupuncture points.

### Perception of Treatment Allocation

To assess the double-blinding of the needles, the patients in the two treatment groups were asked: “Which treatment do you think you have received (active or placebo acupuncture)?” Likewise, the acupuncturists were asked: “Which treatment do you think you have administered (active or placebo acupuncture)?”

In previous studies of penetrating and non-penetrating needles, participants have been able to state whether they believed the needle was penetrating, non-penetrating, or unidentified (e.g. [13]). In our pilot testing, however, all participants chose the unidentified option, and therefore we replaced it with the following question “How confident are you in your answer (identification of active vs. placebo acupuncture)?; “1” = certain, “2” = uncertain, and “3” = unidentified.

### Clues for Perceived Treatment Allocation

To assess if *de qi* influenced the identification of penetrating vs. non-penetrating needles, the patients were asked immediately after application of the needle: “Did you feel *de qi*?”; (yes/no), and acupuncturists were asked right after the needle was removed: “Did you feel *de qi*?”; (yes/no). To patients, the sensation of *de qi* was described as soreness, numbness, heaviness, and distention at the site of needle placement or spreading to other parts of the body. To the acupuncturist, the sensation of *de qi* was described as heaviness or tenseness on the needle he or she was stimulating [18]. As such *de qi* is a subjective sensation.

The patients’ levels of acute pain intensity were measured using the M-VAS [28]. The M-VAS was anchored “no pain sensation” to the left and “the most intense pain sensation imaginable” to the right. Patients were instructed how to rate both pain sensation according to standardized written statements described in detail elsewhere [28].

### Procedure

The study took place at the Department of Dentistry, Aarhus University, Denmark. Prior to the study, a research assistant (SB) informed the patients about the study and introduced them to the pain rating scales. The surgery started at 9 a.m. Immediately after surgery, the patients were asked to rate their acute pain intensity, after which the research assistant wearing a white coat escorted them to an examination room at the research clinic. The patients’ acute pain intensity was rated every 15 minutes. No external stimuli other than orange juice and ice cream were allowed during the study. Patients were informed that they would be randomized to active or placebo acupuncture, and subsequently they drew a number that corresponded to a sealed envelope containing one of these treatments (the treatment group could not be recognized by handling the sealed envelope).

**Fig 3:**
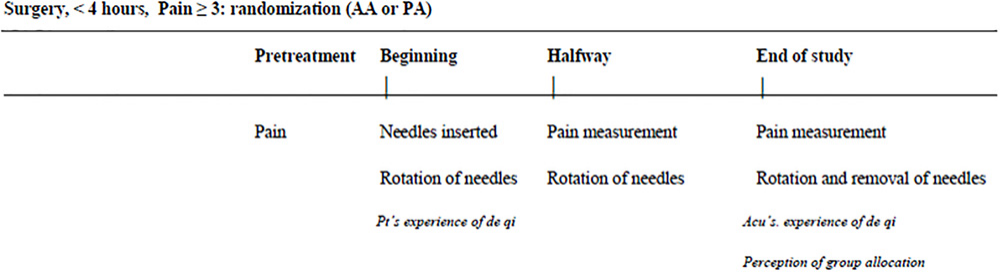
Study design. Patients who developed pain levels of ≥ 3 (0–10) up to 4 hours following surgical removal of one mandibular third molar were randomized to receive active acupuncture (AA) or placebo acupuncture (PA) for 30 minutes. Pain levels were measured at pretreatment, halfway through the session, and at the end of the session. Needles were rotated during insertion, halfway through the session, and at the end of the session right before they were removed. The patients and the acupuncturist were asked about the experience of *de qi* right after insertion of the needle and at the end of the study, respectively. Both the patients and the acupuncturist reported perceived treatment allocation at the end of the study.

The acupuncturists introduced him- or herself to the patients, established rapport, and invited the patients to lie down comfortably in a horizontal dental chair. The research assistant then asked the patients about their current acute pain intensity. Next, the acupuncturist palpated the patients, applied the needles in the designated points and rotated the needles manually for approximately 10 seconds. Immediately after the needles had been applied and the acupuncturist had left the room, the patients were asked if they felt *de qi*. Halfway through the session and at the end of the session, the research assistant again asked the patients about their current acute pain intensity and subsequently the acupuncturist rotated the needles manually. Immediately after the needles were removed, the acupuncturist left the room and answered whether they experienced *de qi* or not. Also, both the patients and the acupuncturists were asked which treatment they believed they had received/administered (active or placebo acupuncture) as well as their level of confidence in the answer (Fig. 3). The acupuncturists were only in the examination room at the onset, halfway through, and at the end of the study.

### Statistical analyses

To test whether the two groups (active and placebo acupuncture) were comparable with respect to gender, ethnicity (European and Asian descent), work status (receiving public benefit, unskilled worker, student, holding a 3-year Bachelor’s degree, or a 5-year Master’s degree, and the surgeon removing the third molar (dental student or oral and maxillofacial surgeon/experienced dentist), chi-square analyses were conducted. To test whether the two groups were comparable with respect to age, dose of preoperative analgesia, dose of intraoperative analgesia, and time until development of acute pain intensity one-way analyses of variance (ANOVA) were conducted with group as factor and the remaining variables as dependent variables.

To test whether the perceived group allocation (active or placebo acupuncture) depended on the actual group allocation (active or placebo), Fisher’s exact tests were conducted for both patients and acupuncturists. To test if the acupuncturists influenced the outcome, the fisher exact test was repeated including stratification by acupuncturists. As an exact test is not available for this statistics, a permutation distribution of the test statistics was conducted to ensure a valid p-value [29]. To test possible differences in the level of confidence when perceiving active or placebo acupuncture, a Mann Whitney test was performed with confidence level (“1” = certain, “2” = uncertain, “3” = unidentified) as the dependent variable and perceived treatment allocation as the independent variable. This test was conducted for both patients and for acupuncturists.

To assess the presence of clues that could influence the identification of active and placebo acupuncture treatment, a general log-linear model was used. For patients, the factors included were: patients’ perception of treatment allocation (active vs. placebo), actual treatment allocation (active vs. placebo), the patients’ experience of *de qi* (yes vs. no), and the patients’ acute pain level (median split of high > 4 vs. low ≤ 4). For acupuncturists, the factors included were: acupuncturists’ perception of treatment allocation (active vs. placebo), actual treatment allocation (active vs. placebo), and acupuncturists’ experience of *de qi* (yes vs. no). An initial model with main effects and all two-way interactions were used and it was in all cases non-significant. The next step in the analyses was to remove all non-significant interactions whereby significant associations were located. The SPSS program version 21 was used, and p < 0.05 was considered statistically significant. The data are expressed in mean values ± SD. “Data are available upon request to the corresponding author”.

## Results

### Characteristics of Patients and Surgery

Almost all patients were Caucasian (97%), and there was a close to equal distribution of males and females (females: 53.7%). The majority of the patients were students (56.7%), and the average age was 25.8 (5.0) years. The average dose of the local analgesia prior to surgery was 4.36 (1.0) ml and the average intraoperative dose was 2.69 (1.4) ml. The average time to development of acute pain intensity ≥ 3 was 57.5 (31.5) minutes.

Three patients did not develop acute pain (≥ 3) 4 hours after the surgical procedure and they were therefore not included in the study. Thus, 67 patients were assigned to either the active or the placebo group and none of these patients were subsequently excluded. There were no significant between-group differences for gender, age, ethnicity, work status, category of surgeon, dose of local analgesia prior to the surgery, dose of local analgesia intraoperatively, time to development of acute pain intensity (all p-values > 0.115). Thus, the two groups can be considered comparable.

### Actual vs. Perceived Treatment Allocation

For patients, perceived treatment allocation depended on actual group allocation (p = 0.015). Sixty-one percent of the patients who perceived the treatment as active acupuncture and 68% of the patients who perceived the treatment as placebo acupuncture identified the treatment allocation correctly (Table 1). There were no significant differences between the patients’ ability to identify active and placebo acupuncture correctly (p = 0.611). In addition, the difference in the patients’ level of confidence when perceiving active or placebo acupuncture did not reach statistical significance (U (36,30) = 44; p = 0.073). For acupuncturists, perceived treatment allocation also depended on actual group allocation (p < 0.001). Eighty-three percent of the acupuncturists who perceived the treatment as active acupuncture identified the treatment allocation correctly, and 81% of the acupuncturists who perceived the treatment as placebo acupuncture identified the treatment allocation correctly (Table 2). There was no significant difference between the acupuncturists p< 0.0004. Also, there were no significant differences between active and placebo acupuncture with respect to perceiving the treatment allocation correctly (p = 0.529). Furthermore, there was no significant difference in the acupuncturists’ level of confidence when perceiving active or placebo acupuncture (U (37,30) = 452; p = 0.137).

**Table 1:** Patients’ perceived treatment allocation in relation to actual treatment allocation.

		Perceived treatment allocation	
		Perceived as “active acupuncture”	Perceived as “placebo acupuncture”
**Actual treatment allocation**	**Active acupuncture**	**22 (61%)**	**10 (32%)**
	**Placebo acupuncture**	**14 (39%)**	**21 (68%)**

**Table 2:** Acupuncturists’ perceived treatment allocation in relation to actual treatment allocation.

		Perceived treatment allocation	
		Perceived as “active acupuncture”	Perceived as “placebo acupuncture”
**Actual treatment allocation**	**Active acupuncture**	**25 (83%)**	**7 (19%)**
	**Placebo acupuncture**	**5 (17%)**	**30 (81%)**

### Clues for Perceived Treatment Allocation

For patients, the only significant association found was between the actual treatment group and the experience of *de qi* (Likelihood Ratio (LR) = 14.231, df = 6, p = 0.027). It may be surprising that there was no significant association between actual and perceived treatment allocation as found in the primary analyses. To further explore this aspect, the patients were divided into those who experienced *de qi* and those who did not, and the relationship between actual and perceived treatment allocation was tested for each group. A statistically significant association between actual and perceived treatment allocation was only found for the 25 patients who experienced *de qi* (LR = 3.869, df = 1, p = 0.049). For acupuncturists, the association between actual and perceived treatment allocation (LR = 27.595, df = 10, p = 0.002) and the association between perceived treatment allocation and the experience of *de qi* (LR = 21.703, df = 11, p = 0.027) were significant. All other associations were not significant.

Post hoc it was noted that 50% of the patients who perceived the treatment as active acupuncture treatment reported *de qi*, whereas 23% of the patients who perceived the treatment as placebo acupuncture reported *de qi*.

## Discussion

Both acupuncturists and patients were in most cases able to identify whether active or placebo acupuncture was administered, indicating that the needles were not fully double-blinded in the present study. The experience of *de qi* appeared to be the main factor leading to identification of treatment allocation.

### Patients and Practitioner Blinding

For both patients and acupuncturists perceived treatment allocation depended on actual treatment allocation, thereby indicating that the needles were fully blinded for neither patients nor acupuncturists and hence not double-blinded. Approximately two-thirds of patients and four-fifths of acupuncturists correctly identified treatment allocation. In specific, patients were able to correctly identify 61% and 68% of active and placebo acupuncture, respectively, whereas acupuncturists were able to correctly identify 83% and 81% of active and placebo acupuncture, respectively (Tables 1 and 2). It should be noted, however, that especially for patients these percentages are not far from a 50/50 distribution. These findings are in line with pharmacological studies testing double blindness in clinical trials involving pain patients [2,5,16,30–32].

To give an example, Turner and colleagues investigated if spinal cord injury patients and their study nurses/physicians were able to identify active pharmacological treatment (amiptriptyline) vs. active placebo (benztropine which gives similar side effects) by asking them to identify treatment allocation at the end of the trial [16]. In line with our results, this study found that approximately two-thirds of patients and three-fourths of study nurses/physicians were able to correctly identify treatment allocation. In specific, patients were able to correctly identify 70% and 55% of active and placebo treatments, respectively, whereas the study nurses/physicians were able to correctly identify 73% and 75% of active and placebo treatment, respectively. Turner and colleagues point out that the percentage of correct identification in their study was lower than in the studies typically reported in the literature [30–32]. This is most likely due to the use of active placebos that induced side effects similar to the active treatment, which made it more difficult to distinguish between active and placebo treatment. Our study may also be seen as using an active placebo in so far as the non-penetrating needles give a sensation of the needle and may elicit *de qi* similar to the active treatment. The reason why the acupuncturists were slightly better than the study nurses/physicians to identify the treatment may be that needle handling gives more direct clues than pill administration.

In our study as well as in the study by Turner et al. [16], there was no significant difference in the level of confidence when identifying active vs. placebo treatment, which suggests that patients and healthcare providers find it equally easy or difficult to identify active and placebo treatments. Noteworthy, both our study and the study by Turner et al. were part of a larger study testing the efficacy of active acupuncture and amitriptyline, respectively [19,33]. In these studies, the active treatments were not significantly more effective than the placebo treatments. Hence, although the blinding was not optimal in these studies, it did not lead to a statistically significant preference of the active over the placebo treatment. It is especially relevant to note that in the larger trial from which the current data are obtained [19], active acupuncture did not significantly reduce pain levels compared with placebo acupuncture when looking at the actual treatment allocation. Yet, looking at patients’ perceived treatment allocation, active acupuncture did significantly reduce pain levels (cf. the short summery of the trial at pages 4 and 5). Thus, although the results of the current study showed that the needles were not fully double-blinded, the patients’ perception of the treatment influenced the clinical treatment outcome (i.e. pain reduction) to a higher extent than the actual treatment.

### Clues to Perception of Treatment Allocation

In order to understand some of the factors which could contribute to correct treatment identification, *de qi* and pain levels were assessed. For patients, there was a significant association between actual treatment allocation and the experience of *de qi*. Also, the overall association between actual and perceived treatment allocation appeared to be driven by the patients who had experienced *de qi*. This finding suggests that for patients, the experience of *de qi* was one of the main clues leading to identification of treatment allocation and thus to unblinding of the treatment.

For acupuncturists there was a significant association between perceived treatment allocation and the experience of *de qi*. In addition, a significant association between actual and perceived treatment allocation was found, indicating that clues in the actual treatment condition, not identified in our study, contributed to breaking the blinding besides the experience of *de qi*.

It may be considered controversial whether acupuncturists can experience *de qi* [18]. Traditionally, *de qi* refers to the excitation of *qi* According to Traditional Chinese Medicine, both the administering acupuncturist and the patient may be able to detect signs of *de qi* [18]. In recent years, however, researchers have put more weight on the patients’ rather than the acupuncturist’ experience during needling, which may at least in part be due to new types of acupuncture, e.g. electro-acupuncture, where the stimulation is not delivered manually by the acupuncturist [18]. Hence, it is well-known from the literature and clinical practice that both patients and acupuncturists may experience *de qi*. It is important to note that in our study the patients and the acupuncturist were not aware of each other’s evaluation of whether *de qi* was experienced or not.

It can be speculated that the relationship between *de qi* and perceived treatment allocation is circular. Hence by telling patients about *de qi* one may influence whether they experience *de qi* and the patients who experience *de qi* may automatically assume that they have received active acupuncture. However, it is important to note that in the current study patients were not given verbal suggestions to the effect that they would experience *de qi*. They were simply informed about *de qi* and asked in a neutral manner whether they experienced *de qi* or not. To ask patients about *de qi* is the standard practice in randomized clinical trials of acupuncture [18]. Yet, in the current study it was found that 50% of the patients who perceived the treatment as active acupuncture reported *de qi* and 23% of the patients who perceived the treatment as placebo acupuncture reported *de qi*. Thus, it does not appear that all the patients’ who experienced *de qi* automatically assumed that they received active acupuncture. Still, given the current design it is difficult to deduce to which extent the experience of *de qi* is influenced by the verbal suggestions related to the description of *de qi* and to which extent it is influenced by needle application. Hence, it is important to be aware that both factors may influence patients reported experience of *de qi*.

Importantly, not only *de qi* contributed to breaking the blinding. It is possible that subtle non-verbal cues transmitted from the acupuncturist to the patients could play an important role as well or even underlie the findings related to *de qi*. Gracely and colleagues have shown that clinicians’ knowledge of the range of possible treatments (placebo or naloxone versus placebo or naloxone or fentanyl) may be transmitted to the patients in conventional double-blind studies, presumably in subtle non-verbal ways [34]. It is possible that a similar phenomenon is taking place in the current study. Hence, during needle application, acupuncturists may be able to correctly identify treatment allocation, possibly due to extensive manipulation of the needle (see below), and this perception may be transmitted to patients in subtle non-verbal ways, thereby leading them to correctly identify treatment allocation. This hypothesis is in agreement with the finding that acupuncturists to a higher extent than patients correctly identified treatment allocation, although more subtle research designs are necessary to explore this further.

In so far as the questions concerning perceived treatment allocation were posed at the end of the session as it is often done in test of double blindness [4], it could be speculated that patients’ perception of treatment allocation was influenced by the experience of acute pain [16]. In other words, patients who had high levels of acute pain at the end of the session perceived the treatment as placebo acupuncture, whereas patients who had low levels of acute pain perceived the treatment as active acupuncture. This possibility was tested, but there was no association between patients’ level of acute pain and perceived treatment allocation.

### Penetrating and Non-Penetrating Needles

The finding that the penetrating and non-penetrating needles were not fully blinded in the current study is in contrast to previous studies using these needles [13–15]. The different findings may be explained by methodological differences. In the previous studies, acupuncturists typically administered combinations of penetrating/penetrating, penetrating/non-penetrating and non-penetrating/non-penetrating into a single acupuncture point (e.g. [12]), whereas in the present study acupuncturists administered the same needles, e.g. 5 penetrating or 5 non-penetrating needles, into 5 different acupuncture points, respectively. Also, in the previous studies, acupuncturists were able to report whether they believed the needle was penetrating, non-penetrating, or unidentified (e.g. [13]), but in our study they were forced to choose between penetrating or non-penetrating needles and subsequently rate their level of confidence in the assessment. Importantly, our study involved patients as opposed to healthy volunteers and the acupuncture treatment was performed according to the Chinese tradition of acupuncture in which needle manipulation during needle insertion is a central element, which is not the case in Japanese acupuncture [18,35], in which the needles are developed and previously tested [13]. Hence, the more extensive manipulation of the needles in our study (rotation at the beginning, halfway through, and the end of the study as well as insertion into several points) may have contributed to the reported experience of *de qi* and thereby to the breaking of the double-blinding. Thus, the results may not only reflect the ability to blind acupuncture treatment but also the influence of different cultures style on acupuncture [18] and it would therefore be relevant to test the needles in a randomized clinical trial performed within Japanese style of acupuncture.

Still, although the needles were not fully double-blinded, approximately one-third of the patients and nearly one-fifth of the acupuncturists were not able to correctly identify treatment allocation (Table 1). In most studies, sham acupuncture is performed by superficial needling at non-acupuncture points (e.g. [36]), by penetrating needling at non-acupuncture points (e.g. [6]), or by placebo needles (e.g. [8,10]) that are identifiable to the acupuncturist [9]. Hence, the penetrating and non-penetrating needles are so far the only needles that allow for some degree of practitioner blinding. Knowing which treatment is administered is probably not the same as identifying treatment allocation correctly at the end of a trial [3], so the possibility of applying a needle that can be blinded to some extent is likely to improve the validity of a study.

### Conclusion and Future Directions

It is difficult to fully double-blind both non-pharmacological treatments like acupuncture and pharmacological treatments. As the breaking of the double-blinding may hamper the conclusions that can be drawn from studies, it is important to pay attention to this issue [4]. As long as double blinding is not a reality, it is possible that, for example, acupuncture studies will find differences between active and placebo treatments that may erroneously be interpreted as an effect of the active treatment, which is in reality a non-specific placebo effect. Different strategies for improving double-blinding have been attempted, but our study shows that new approaches are needed. Although more advanced placebos that could improve the double-blinding may be developed, it may also be helpful to think about new approaches. Both expectations and emotional feelings have been shown to account for large amounts of the variance in placebo effect in acupuncture and pharmacological treatments [19,37], so an additional strategy may also be to assess the placebo component of treatments via these factors [38,39]. Still, until new strategies have been developed, it seems warranted to always ask patients and acupuncturists/healthcare providers to identify treatment allocation in order to understand to which extent unblinding may have contributed to the obtained results.
